# Changing the definition of treatment success alters treatment outcomes in periprosthetic joint infection: a systematic review and meta-analysis

**DOI:** 10.5194/jbji-9-127-2024

**Published:** 2024-04-26

**Authors:** Eytan M. Debbi, Tyler Khilnani, Ioannis Gkiatas, Yu-Fen Chiu, Andy O. Miller, Michael W. Henry, Alberto V. Carli

**Affiliations:** 1 Department of Orthopedic Surgery, Adult Reconstruction and Joint Replacement, Hospital for Special Surgery, New York, NY, USA; 2 Biostatistics Core, Research Administration, Hospital for Special Surgery, New York, NY, USA; 3 Department of Infectious Disease, Hospital for Special Surgery, New York, NY, USA

## Abstract

**Background**: Variability in the definition of treatment success poses difficulty when assessing the reported efficacy of treatments for hip and knee periprosthetic joint infection (PJI). To address this problem, we determined how definitions of PJI treatment success have changed over time and how this has affected published rates of success after one-stage and two-stage treatments for hip and knee PJI. **Methods**: A systematic review following Preferred Reporting Items for Systematic Reviews and Meta-Analyses (PRISMA) guidelines was conducted to identify one-stage and two-stage revision hip and knee PJI publications in major databases (2006–2021). Definition of treatment success, based on Musculoskeletal Infection Society tier criteria, was identified for each study. Publication year, number of patients, minimum follow-up, and study quality were also recorded. The association of success definitions and treatment success rate was measured using multi-variable meta-regression. **Results**: Study quality remained unchanged in the 245 publications included. Over time, no antibiotics (tier 1) and no further surgery (tier 3) (40.7 % and 54.5 %, respectively) became the two dominant criteria. After controlling for type of surgery, study quality, study design, follow-up, and year of publication, studies with less strict success definitions (tier 3) reported slightly higher odds ratios of 1.05 [1.01, 1.10] ( p=0.009 ) in terms of treatment success rates compared to tier 1. **Conclusions**: PJI researchers have gravitated towards tier-1 and tier-3 definitions of treatment success. While studies with stricter definitions had lower PJI treatment success, the clinical significance of this is unclear. Study quality, reflected in the methodological index for non-randomized studies (MINORS) score, did not improve. We advocate for improving PJI study quality, including clarification of the definition of treatment success.

## 1 Introduction

Periprosthetic joint infection (PJI) is a rare but devastating complication that can occur following total joint arthroplasty (Carli et al., 2019). Although the last decade has brought forward international consensus recommendations and completion of multi-center randomized control trials (Osmon et al., 2013; Parvizi et al., 2011, 2018; Zmistowski et al., 2014), the outcomes of PJI treatment have not improved meaningfully (Xu et al., 2020) and continue to be a source of substantial morbidity (Goel et al., 2018) and mortality (Lum et al., 2018). Such stagnation in treatment improvement has led to publications investigating the role of implant retention (Deng et al., 2021), long-term antibiotic suppression (Siqueira et al., 2014), and an unsurprising departure from adhering to guideline-recommended treatments (Armstrong et al., 2018). Furthermore, two-stage exchange arthroplasty, long considered to be the gold-standard treatment for chronic PJI, has recently been challenged by one-stage exchange, which has expanded from a single-center experience (Zahar et al., 2016, 2019) to being adopted by multiple centers across the world (Kildow et al., 2020; Klemt et al., 2021; Marmor et al., 2020; Negus et al., 2017).

A critical variable that interferes with the determination of the relative efficacy of PJI treatments is the definition of treatment success. Unfortunately, the PJI literature has been historically inconsistent in this regard. The time interval prior to complications (Diaz-Ledezma et al., 2013), whether septic- or aseptic-related revision surgery is performed, and the use of antibiotic suppression are examples of key descriptors that can radically alter how success with PJI treatment is achieved. As such, a work group from the Musculoskeletal Infection Society (MSIS) sought to define successful PJI management, defining tiers of outcomes for the treatment for PJI, as summarized in Table 1 (Fillingham et al., 2019). The impact of this new tiered-outcome tool is evident in the PJI literature, having been cited over 20 times in under 2 years since publication. Yet, to date, no comprehensive appraisal of how the PJI literature has defined treatment success exists, and no group has analyzed how the definition of treatment success affects reported outcomes.

**Table 1:** Definition of success following treatment for periprosthetic joint infection (PJI) by tier, modified from Fillingham et al. (2019).

Tier	Definition of prosthetic joint infection (PJI) treatment success
Tier 1	No further surgery; no suppressive antibiotic therapy
Tier 2	No further surgery; antibiotic suppressive therapy permitted
Tier 3A	No septic revision surgery, but aseptic revision over 1 year post-initiation of PJI treatment is permitted
Tier 3B	No revision surgery within 1 year post-initiation of PJI treatment, but septic or aseptic revision after 1 year are permitted; no salvage-type procedures (arthrodesis, resection arthroplasty, amputation)
Tier 3C	No septic revision surgery within 1 year post-initiation of PJI, but aseptic revision surgery within 1 year is permitted; no salvage-type procedures (arthrodesis, resection arthroplasty, amputation)
Tier 3D	No salvage-type procedures (arthrodesis, resection arthroplasty, amputation)
Tier 3E	No spacer retained; salvage-type procedures (arthrodesis, resection arthroplasty, amputation) are permitted
Tier 3F	Spacer may be retained
Tier 4A	No death within 1 year from initiation of PJI treatment
Tier 4B	Survival analysis only

The goals of the present study were to conduct a systematic review to determine how the definition of PJI treatment success has changed over time and to apply a statistical evaluation of how the MSIS working group tiers influence treatment outcomes for PJI. The MSIS group tiers in Table 1 have different criteria for the outcomes of successful infection treatment, with tier 1 being the least encompassing and tiers 3 and 4 being more encompassing. Since less encompassing tiers limit the number of studies meeting a certain definition of success, lower tiers, such as tier 1, have a stricter definition of success. We hypothesized that stricter definitions of PJI treatment success are being used over time, and the use of stricter definitions of PJI treatment success will confer significantly lower treatment success rates.

## 2 Materials and methods

### 2.1 Search criteria

We followed the Preferred Reporting Items for Systematic Reviews and Meta-Analyses (PRISMA) guidelines to query the US National Library of Medicine (PubMed/MEDLINE), the EMBASE, and Cochrane databases for publications from 1 January 2006 through 31 December 2021 regarding revision following PJI utilizing specific keywords (see Tables S1 and S2 in the Supplement). To focus on treatment outcomes specifically for chronic PJI, only publications that evaluated the outcomes after one- or two-stage revision of total hip or knee arthroplasty (THA or TKA) for PJI were included. The 15-year period (2006–2021) was chosen to encompass the entirety of the published literature regarding antibiotic suppression and PJI and to include research conducted before and after international recommendations pertaining to the diagnosis and management of PJI were published (Osmon et al., 2013; Parvizi et al., 2011, 2018; Zmistowski et al., 2014).

### 2.2 Inclusion and exclusion criteria

Inclusion criteria were agreed upon by the authors before the search was performed. Studies were retained for analysis if they were written in English, involved human subjects over the age of 18, investigated treatment outcomes of single-stage and/or two-stage hip or knee exchange arthroplasty for PJI, and explicitly provided both a diagnostic definition for PJI and a definition for treatment success. Case reports, preclinical studies, narrative or systematic reviews, articles that investigated implant retention treatments, and articles not available as full texts were excluded.

### 2.3 Data collection and stratification

Article titles and abstracts were independently reviewed and graded by three authors (Ioannis Gkiatas, Tyler Khilnani, Eytan M. Debbi) for the criteria necessary to determine the methodological index for non-randomized studies (MINORS) score and tier classification. Qualifying full-text articles were retrieved and independently evaluated through the application of inclusion and exclusion criteria. Any discrepancy between authors for study inclusion was settled by the senior author (Alberto V. Carli). For each qualifying study title, the author, year published, study design, number of patients, number of joints, type of surgical treatment, duration of follow-up, definition of success, and reported treatment success rate were collected.

Articles were categorized into tiers based on how the authors defined treatment success. We used the MSIS work group guidelines (Fillingham et al., 2019) of the definition of treatment success in each tier (Table 1). Specifically, tier 1 includes patients with infection control without antibiotic suppression; tier 2 includes patients with infection control but requiring antibiotic suppression; tier 3 is subdivided into 3A–F and includes patients requiring reoperation, revision, or spacer retention; and tier 4 includes patients who have died after treatment for PJI.

### 2.4 Assessment of study quality

Publication bias was assessed by creating a funnel plot, a graphical tool to show the study effect size against its precision and using the Egger test for asymmetry. Between-study heterogeneity was tested using the $\chi^{2}$ test and quantified using the $I^{2}$ statistic. $I^{2}$ values < 25 %, 25 %–75 %, and > 75 % were considered to be low, moderate, and high, respectively (Stuart et al., 2015). To assure low risk of bias in the qualifying studies, each reviewer independently scored each study using the methodological index for non-randomized studies (MINORS) criteria (Slim et al., 2003) following the guidelines established by Slim et al. (2003). While used for non-randomized studies, the MINORS criteria also have additional measurements to be used for randomized trials as well. Due to the heterogeneity of the literature, we calculated the mean for cohort studies and randomized control trials (RCTs) separately and compared the median MINORS score for studies using definitions of success in each tier.

### 2.5 Statistical analysis 

The primary outcome measure was treatment success rate in each tier. Random-effect models were used to pool complementary treatment success proportional estimates post-PJI. Between-study variance was estimated using the restricted maximum likelihood method. Multivariable meta-regression was used to assess the potential association of success definitions and treatment success rate, accounting for the type of surgery (one stage versus two stage), MINORS score, type of study design (cohort or RCT), and year of publication. Using multivariate regression, we calculated the odds ratios (ORs) for success to compare the likelihoods of successful PJI treatment outcomes accounting for the tier of success definition, type of surgery (one or two stage), MINORS score (risk of bias as a measure of publication quality), study design (cohort or RCT), duration of follow-up, and year of publication. All analyses were performed using the metafor and meta packages implemented in RStudio software version 1.4.1717 (RStudio, PBC, Boston, MA).

## 3 Source of funding

There are no sources of funding to declare.

## 4 Results

### 4.1 Search results

**Figure 1:**
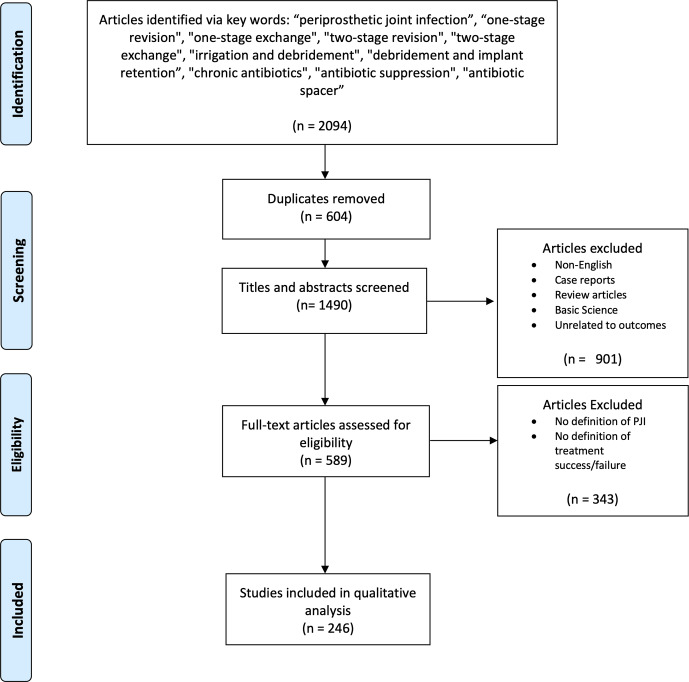
PRISMA flow diagram of record search, screening, and selection. Adapted from Moher et al. (2009).

A total of 2094 studies were identified (515 one stage and 1579 two stage), of which 245 met the necessary criteria (Fig. 1). We extracted data from 31 studies reporting on one-stage exchange, 181 studies reporting on two-stage exchange, and 33 studies reporting on both one-stage and two-stage treatment for hip or knee PJI.

### 4.2 Study quality

**Figure 2:**
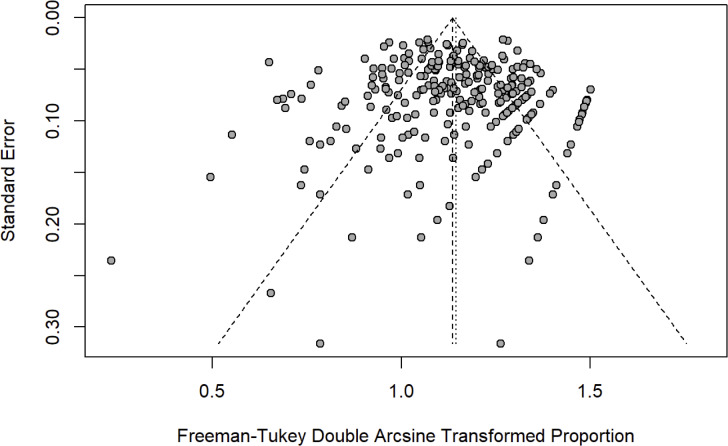
The points, each representing one study, clustered at the top of the funnel plot indicating low level of publication bias in the study (Egger's test, p=0 .276).

**Figure 3:**
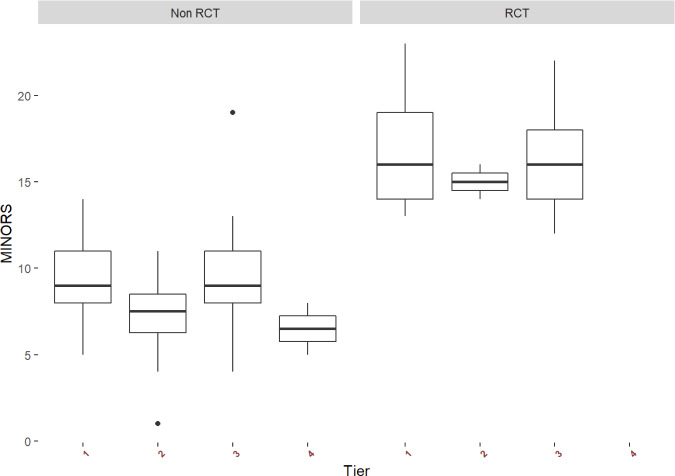
Methodological index for non-randomized studies (MINORS) score for all non-randomized controlled studies (non-RCT) and randomized controlled studies (RCT) of hip and knee periprosthetic joint infection studies using different tiers of definition of success.

An a priori funnel plot and the Egger test ( p=0 .277) did not identify publication bias prior to completion of the meta-analysis (Fig. 2). Between-study heterogeneity was high ( $I^{2}$ 84.5 % [82.9 %, 86 %], p<0.0001 ). The median MINORS criteria score was 9/16 (range 1–14) for cohort studies and 16/24 (range 12–23) for randomized controlled trials, indicating low risk of bias. Over time, there were no significant changes in average MINORS scores (Fig. 3). When examining non-RCT studies and RCT studies separately, MINORS scores were similar across studies with different tiers (different definitions of success) (Fig. 3).

### 4.3 Definition of success of PJI treatment by tier 

**Figure 4:**
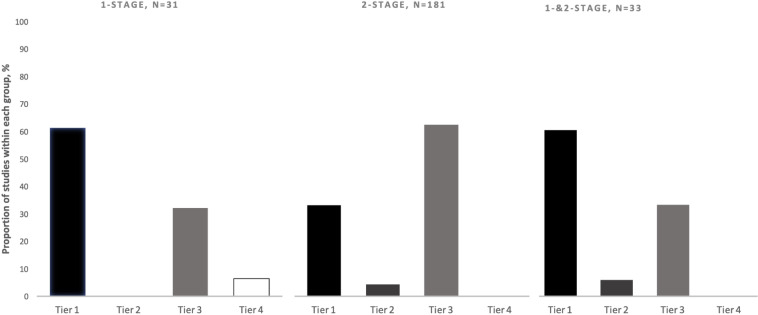
Proportion of hip and knee periprosthetic joint infection (PJI) studies reporting outcomes of one-stage, two-stage, or one- and two-stage revisions categorized by the definition of success used (tiers 1–4; see Table 1).

**Figure 5:**
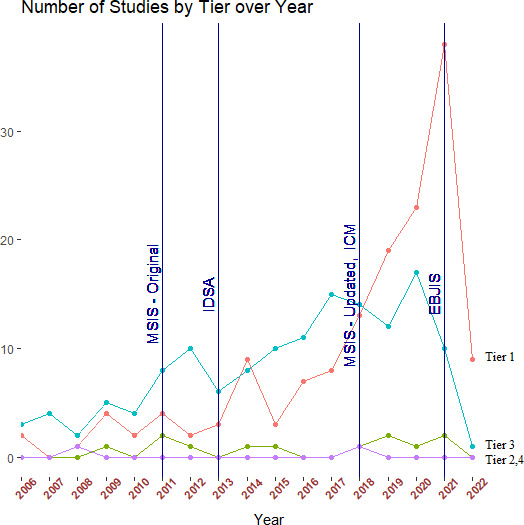
Number of hip and knee periprosthetic joint infection studies using each tier of definition of treatment success over time. Year of publication of infection diagnostic criteria according to the Musculoskeletal Infection Society (MSIS), the Infectious Disease Society of North America (IDSA), and the first and second International Consensus Meeting for periprosthetic joint infection (first and second ICM) are marked.

As predicted, the definition of success used was not uniform across the PJI literature. Approximately 40 % of included studies utilized a tier-1 definition (no further requirement for antibiotics) of PJI treatment success, 4 % utilized tier 2 (no further surgery, antibiotic suppression permitted), 55 % utilized tier 3 (no further surgery), and < 1 % utilized Tier 4 (death). Of the 134 tier-3 studies, the most commonly utilized subgroup definition was tier 3c (no septic revision within 1 year, aseptic revision permitted) (72 %). The tier-1 success definition was used more often in studies reporting one-stage treatment outcomes (61 %) as compared to studies reporting two-stage treatment outcomes (33 %) (Fig. 4). The tier-3 definition was more commonly used in studies reporting two-stage treatment outcomes (62 %) rather than one-stage treatment outcomes (32 %) (Fig. 4). From 2006–2020, the number of studies using tier-1 and tier-3 definitions of success increased, while those using tier-2 and tier-4 definitions remained low (Fig. 5). While tier-1 and tier-3 definitions remained the most commonly reported primary outcomes over time, neither became dominant at any time point.

### 4.4 Meta-analysis: does success definition affect rate of success?

The weighted mean success rate (95 % CI) in studies reporting one-stage treatment outcomes was 0.84 [0.80, 0.88] based on the tier-1 success definition, 0.59 [0.25, 0.89] based on the tier-2 success definition, 0.96 [0.93, 0.99] based on the tier-3 success definition, and 0.94 [0.86, 0.99] based on the tier-4 success definition. In two-stage studies, the mean success rate was 0.82 [0.79, 0.84] based on the tier-1 success definition, 0.83 [0.79, 0.87] based on the tier-2 success definition, and 0.82 [0.84, 0.86] based on the tier-3 success definition (see Appendix).

ORs were calculated to determine the likelihood of successful PJI treatment. Among studies reporting outcomes of one-stage PJI surgery, a tier-3 definition of success significantly increased the odds of success compared to the use of a tier-1 definition (OR 1.16, p=0.002 , Table 2). With both types of procedures considered together, the odds ratio was also higher when studies used a tier-3 rather than tier-1 definition of success (OR 1.05, p=0.009 , Table 2), but among studies reporting only two-stage surgery outcomes, the odds of success were similar regardless of the definition of success. Tier-2 or tier-4 definitions of success, MINORS score (risk of bias as a measure of publication quality), study design (cohort or RCT), duration of follow-up, and year of publication did not significantly affect the odds of success.

**Table 2:** Effect of success definition on reported prosthetic joint infection (PJI) treatment outcome^a^.

	Odds ratio	95 % confidence		P value
		interval		
One-stage studies				
Tier 2 (vs. tier 1)	0.77	0.56	1.06	0.113
Tier 3 (vs. tier 1)	1.16	1.06	1.28	0.002^**^
Tier 4 (vs. tier 1)	1.21	0.97	1.52	0.097
Two-stage studies				
Tier 2 (vs. tier 1)	0.97	0.87	1.09	0.635
Tier 3 (vs. tier 1)	1.03	0.99	1.08	0.115
All studies				
Tier 2 (vs. tier 1)	0.97	0.87	1.08	0.554
Tier 3 (vs. tier 1)	1.05	1.01	1.10	0.009^**^
Tier 4 (vs. tier 1)	1.13	0.91	1.42	0.274
Two stage (vs. one stage)	0.95	0.90	0.99	0.030^**^

^a^ Odds ratios were calculated as described in the “Materials and methods” section to compare the likelihood of treatment success in studies using different tiers of success definition, accounting for study follow-up, year of publication, study randomization, and MINORS score.

## 5 Discussion

Although the optimal treatment for PJI remains a topic of intense investigation and debate, little attention has been paid to how studies define the success of such treatments. Through analyzing 15 years' worth of qualifying PJI publications based on definitions of success described by the MSIS working group (Fillingham et al., 2019), our study has identified interesting trends within the PJI literature regarding how treatment success is reported. A reassuring finding is that the strictest definition of PJI treatment success (tier 1, no further requirement for antibiotics) has become more commonly utilized over time, possibly reflecting demand from peer-reviewed journals that receive a higher volume of study submissions. However, a concerning additional finding was that PJI study quality, as described by the MINORS score, has not substantially improved over time. Furthermore, despite the increase in publications using a tier-1 success definition, those using a tier-3 success definition, which is less strict, have similarly increased. Our findings suggest that much work still needs to be done by PJI investigators and peer-reviewed journals to standardize the reporting of PJI treatment outcomes and to improve their minimum methodological standards.

Interestingly, publications rarely utilized tier-2 and tier-4 definitions of PJI treatment success. Tier 2 specifically includes patients on suppressive antibiotics, and tier 4 includes mortality. While we expected a more substantial proportion of studies to use tier-2 definitions, this was not found to be the case. This may suggest a reduction in the use of antibiotic suppression or inconsistent reporting of these patients in the literature. Instead, most PJI studies appear to have divided their results into either tier-1 or tier-3 definitions of success, and a number of this review's comparisons involve tier-1 outcomes compared to tier-3 outcomes. Focusing on this comparison, we observed that a tier-3 definition significantly increased the odds of PJI treatment success, and this confirmed our hypothesis that using stricter definitions of success leads to lower reported success rates. However, the odds ratio difference between tier-1 and tier-3 outcomes was not very large (OR of 1.05 for all studies), likely reflecting that the tiers may not really be so different since the need for revision surgery (the marker of failure for tier 3) inevitably also confers a failure of infection control (the marker of failure for tier 1). Since most studies appear to be using tier-1 or tier-3 definitions of success, we recommend that future PJI studies be required to define these groups clearly. For example, if they choose to report on patients undergoing reoperation after PJI treatment (i.e., tier 3) then these outcomes should be clearly separated from results on patients not undergoing reoperation after PJI treatment (i.e., tier 1). Furthermore, peer-reviewed journals should consider mandating that PJI treatment studies complete necessary methodological checklists (such as STROBE for cohort, case-control, and cross-sectional studies and RECORD for observational studies) to confer appropriate study quality.

Our multi-variable meta-analysis determined that two-stage exchange arthroplasty was associated with a significantly lower rate of treatment success compared to one-stage exchange when accounting for diagnostic definition, treatment definition, and study quality. Although interesting, we do not conclude that such a detected difference confers a true deficiency in the utility of two-stage exchange. This is because numerous confounding variables cannot be accounted for in such a comparison, including selection criteria for one-stage surgery, criteria utilized to justify re-implantation, and the use of antiseptic solutions. Instead, our findings demonstrate the necessity of incorporating treatment success definition and study quality when performing comparative analyses of PJI treatments. The adoption of an editorial standard mandating minimal study quality requirements and reporting outcomes according to the MSIS working-group tiers could be one method of facilitating treatment comparisons.

We acknowledge several limitations in the present study. First, the inherent retrospective nature of the systematic review limits its possible findings to the quality and scope of studies which met inclusion criteria. Second, we acknowledge that not all studies provided explicit definitions of what constituted PJI treatment success, causing us to utilize study methodology, results in tables, and discussion points to interpret the definitions. It is therefore possible that the designated study tier in these cases may not have been as accurate as for other studies which were more explicit in their intentions. Third, we also acknowledge that our summary findings for study quality and diagnostic definitions do not apply to the entirety of the PJI literature but only to those studies examining one-stage and two-stage exchange procedures. Therefore, our statements pertaining to future restrictions on publications may not necessarily apply to PJI studies examining debridement, antibiotic, and implant retention (DAIR) techniques, which make up a sizable portion of the PJI literature. On the other hand, our study has been carefully planned, and our meta-analyses have accounted for a multitude of study variables that could have otherwise skewed results. In addition, as a systematic review, we included all studies, including those with clinical follow-up of less than 2 years. While we acknowledge the limitations of such studies, these studies were purposefully included as the goal of this review was to characterize the PJI literature over 15 years. In addition, we controlled for study quality and follow-up in our data analysis; therefore, we hope the effects of low-quality studies on the results have been minimized. Another limitation of the present study is that only one scoring system was used to assess for study reporting quality, and there may be other, better methods for assessing study quality. We also acknowledge that the MINORS criteria were specifically designed for non-randomized studies, making it difficult to draw comparative conclusions between RCTs over time. MINORS, however, does evaluate pertinent tenets of study design, including appropriate sample size, endpoints, and follow-up; therefore, relevant conclusions can still be made on randomized studies and, consequently, in relation to the remainder of the PJI literature.

In conclusion, our work has defined the specific changes in the definition of PJI treatment success which researchers have used over time. These findings indicate that appropriately defining treatment success is a pertinent goal for which the PJI literature should strive as it influences study outcomes and consequently affects interpreted outcomes in treatment comparisons. Consequently, we believe that clinical PJI studies should explicitly report their definition of treatment success utilizing the MSIS working-group tiers to be published in the peer-reviewed literature.

## Supplement

10.5194/jbji-9-127-2024-supplement The supplement related to this article is available online at: https://doi.org/10.5194/jbji-9-127-2024-supplement.

## Data Availability

Data supporting this study and software coding results are available from the Stavros Niarchos Complex Joint Reconstruction Center Registry at the Hospital for Special Surgery (HSS). Requests for access to the data are subject to review and approval of HSS and may require a data-sharing agreement to address privacy, confidentiality, and ethical concerns.

## Code availability

Data supporting this study are available from the Stavros Niarchos Complex Joint Reconstruction Center Registry at the Hospital for Special Surgery (“HSS”). Requests for access to the data are subject to review and approval of HSS and may require a data sharing agreement to address privacy, confidentiality and ethical concerns.

## Appendix

The supplement related to this article is available online at: https://doi.org/10.5194/jbji-9-127-2024-supplement.
