# Supplementary material for: Changing the definition of treatment success alters treatment outcomes in periprosthetic joint infection: a systematic review and meta-analysis
Source: J Bone Jt Infect. 2024 Apr 26;9(2):127–36. doi: 10.5194/jbji-9-127-2024 (PMC11184615; doi:10.5194/jbji-9-127-2024)
Supplement: The supplement related to this article is available online at: https://doi.org/10.5194/jbji-9-127-2024-supplement. [file jbji-9-127-supplement.pdf]

Supplement of J. Bone Joint Infect., 9, 127–136, 2024
https://doi.org/10.5194/jbji-9-127-2024-supplement
© Author(s) 2024. CC BY 4.0 License.

JBJI
Journal of Bone and Joint Infection
Open Access

Supplement of

Changing the definition of treatment success alters treatment outcomes in periprosthetic joint infection: a systematic review and meta-analysis

Eytan M. Debbi et al.

Correspondence to: Alberto V. Carli (carlia@hss.edu)

The copyright of individual parts of the supplement might differ from the article licence.

Table S1: Search terms for each database regarding the 1-stage revisions

Database	Search Terms
Pubmed/MedLine	periprosthetic joint infection[tw]<br>OR peri-prosthetic joint infection[tw]<br>OR PJI[tw]<br>OR Prosthesis-Related Infections[mesh]<br>AND<br>one-stage revision[tw]<br>OR 1-stage revision[tw]<br>OR single-stage revision[tw]<br>OR one-stage exchange[tw]<br>OR 1-stage exchange[tw]<br>OR single-stage exchange[tw]
Cochrane	"periprosthetic joint infection":ti,ab,kw<br>OR "peri-prosthetic joint infection":ti,ab,kw<br>OR PJI:ti,ab,kw<br>OR [mh "Prosthesis-Related Infections"]<br>AND<br>"one-stage revision":ti,ab,kw<br>OR "1-stage revision":ti,ab,kw<br>OR "single-stage revision":ti,ab,kw<br>OR "one-stage exchange":ti,ab,kw<br>OR "1-stage exchange":ti,ab,kw<br>OR "single-stage exchange":ti,ab,kw
Embase	"periprosthetic joint infection":ti,ab,de,tn,kw<br>OR "peri-prosthetic joint infection":ti,ab,de,tn,kw<br>OR PJI:ti,ab,de,tn,kw<br>OR 'periprosthetic joint infection'/exp<br>AND<br>'one-stage revision'/exp<br>OR "one-stage revision":ti,ab,de,tn,kw<br>OR "1-stage revision":ti,ab,de,tn,kw<br>OR "single-stage revision":ti,ab,de,tn,kw<br>OR "one-stage exchange":ti,ab,de,tn,kw<br>OR "1-stage exchange":ti,ab,de,tn,kw<br>OR "single-stage exchange":ti,ab,de,tn,kw

Table S2: Search terms for each database regarding the 2-stage revisions

Database	Search Terms
Pubmed/MedLine	periprosthetic joint infection[tw]<br>OR peri-prosthetic joint infection[tw]<br>OR PJI[tw]<br>OR Prosthesis-Related Infections[mesh]<br>AND<br>two-stage revision[tw]<br>OR 2-stage revision[tw]<br>OR two-stage exchange[tw]<br>OR 2-stage exchange[tw]
Cochrane	"periprosthetic joint infection":ti,ab,kw<br>OR "peri-prosthetic joint infection":ti,ab,kw<br>OR PJI:ti,ab,kw<br>OR [mh "Prosthesis-Related Infections"]<br>AND<br>"two-stage revision":ti,ab,kw<br>OR "2-stage revision":ti,ab,kw<br>OR "two-stage exchange":ti,ab,kw<br>OR "2-stage exchange":ti,ab,kw
Embase	"periprosthetic joint infection":ti,ab,de,tn,kw<br>OR "peri-prosthetic joint infection":ti,ab,de,tn,kw<br>OR PJI:ti,ab,de,tn,kw<br>OR 'periprosthetic joint infection'/exp<br>AND<br>'two-stage revision'/exp<br>OR "two-stage revision":ti,ab,de,tn,kw<br>OR "2-stage revision":ti,ab,de,tn,kw<br>OR "two-stage exchange":ti,ab,de,tn,kw<br>OR "2-stage exchange":ti,ab,de,tn,kw
